# Citizen science initiatives document biodiversity baselines at an urban lake

**DOI:** 10.7717/peerj.17209

**Published:** 2024-04-17

**Authors:** Alyssah Ahern, Daniel F. Hughes

**Affiliations:** Department of Biology, Coe College, Cedar Rapids, IA, United States of America

**Keywords:** Biodiversity, Baseline data, Urban biodiversity, Global change, Urban lake, Species richness, Community composition

## Abstract

Changes to biodiversity from urbanization are occurring worldwide, and baseline data is vital to document the magnitude and direction of these alterations. We set out to document the biodiversity of an urban lake in Eastern Iowa that was devoid of baseline data prior to a renovation project that will convert the site into a major area for human recreation. Throughout the course of one year, we studied the biodiversity at Cedar Lake utilizing the citizen-science application iNaturalist coupled with semi-structured BioBlitz events, which we compared to previous opportunistic observations at the site. From a semi-structured approach to document biodiversity with citizen science, our analyses revealed more diverse community metrics over a shorter period compared to more than a decade of prior observations.

## Introduction

Global biodiversity is currently experiencing changes that will impact the future makeup of the planet (Sala et al., 2000). For example, the extinction rate of species now greatly exceeds baseline levels (Pimm et al., 2014), which has been attributed to the increasing fragmentation of natural habitats, continued pollution of the environment, and unsustainable consumption of natural resources (Butchart et al., 2010). One of the main drivers of these impacts to biodiversity is urbanization (Piano et al., 2020), and while urbanized areas account for just 3% of the total land use of the planet, the changes wrought from the construction of buildings, roads, and other infrastructure extends far beyond land use (Chapman, 2003; Faeth, Bang & Saari, 2011). Aquatic habitats in cities are particularly vulnerable as they are often hotspots for local biodiversity (Hill et al., 2017), yet also centers for human use, recreation, and waste disposal (Hassall, 2014). Improvements to enhance the value of urban waterbodies and increase their recreational appeal can have both positive and negative impacts on biodiversity. For example, cosmetic changes to an urban lake can provide new habitat features for some species, such as adding a boardwalk where algae can attach and fish can congregate, thereby increasing their abundance and diversity, which can in turn help to raise local awareness of the importance of conserving the waterbody and its surrounding habitat (Savard, Clergeau & Mennechez, 2000; Qiu, Lindberg & Nielsen, 2013). However, urban improvements can also have negative impacts on biodiversity. For example, wetland management practices, even if intended to aid conservation, can unintentionally impact other species, and potentially public health by providing habitat for organisms that may vector diseases (*e.g.*, Hanford, Webb & Hochuli, 2020). Furthermore, urban improvements may lead to increased human disturbances, noise and light pollution, and removal of key habitat elements, which can disrupt the behavior, reproduction, and migration of wildlife (Ewing et al., 2005). To minimize the negative impacts of urban sprawl on biodiversity, it is important for urban planners to consider the resident ecological communities when designing and implementing these projects (*i.e.,* Smart Growth: Daniels, 2001).

Establishing a baseline of the species diversity that exists at a site prior to change provides a point of reference against which future observations can be compared (Mihoub et al., 2017). It can be an insurmountable challenge to accurately track changes in biodiversity without a baseline (Magurran et al., 2010). By comparing current data with such a baseline dataset, we can identify species that are declining, expanding, or shifting their distribution, as well as changes in community processes and ecosystem functions (Gullison et al., 2015). Landscape changes in urban areas often decrease the amount of habitable land available to local biodiversity, and many such initiatives are implemented without much knowledge of the organisms who resided in a habitat prior to changes (Bobrowiec & Tavares, 2017). Temporal baselines are also needed to establish targets for biodiversity conservation and progress to conservation goals to be evaluated. Performing studies to document the biodiversity in habitats before projects take place is important to be able to verify how much of the diversity found its way back to the habitat once the project was completed. Having reliable baseline data is needed to reconstruct the impacts of human activities, climate change, and other factors on biodiversity, and for developing effective strategies to protect and conserve natural ecosystems. Such standard biodiversity monitoring is also needed to identify meaningful benchmarks for biodiversity (Feest, 2006).

Large ecological datasets are critical to track changes in biodiversity but are logistically challenging to cover the spatial and temporal scales needed for understanding the magnitude of an impact (Costello & Wieczorek, 2014). Citizen science is a cost-effective, rapid, and efficient way to gather data on biodiversity over large areas and long intervals, which can be leveraged for documenting changes in biodiversity (Theobald et al., 2015). In recent years, the use of citizen science has become increasingly popular for monitoring and recording changes in the natural world (Chandler et al., 2017). Online applications such as iNaturalist allow citizens to engage with nature by taking photos of organisms they encounter and upload them to a site for other members of the community to identify. However, there are some limitations to the use of citizen science for documenting changes in biodiversity as the quality of the data may be unreliable, or there may be biases due to the locations where people choose to collect data, or certain species may be considered more interesting to document (Tweddle et al., 2012). Regardless, such biodiversity platforms are the increasingly becoming the sources of data for understanding changes in ecological communities over time, informing conservation efforts, and documenting impacts on biodiversity (*e.g.*, Kirchhoff et al., 2021).

Our study focused on Cedar Lake in Cedar Rapids of eastern Iowa, which is a small, urban lake that is frequently used by people for recreational fishing and has a documented history of pollution. The City of Cedar Rapids enacted a 5-year improvement plan to bolster the flood wall and increase the recreational-use of the lake, with construction starting in 2022. Consequently, in 2021, we set out to create a spatially explicit, temporal baseline of biodiversity data at Cedar Lake through the use of semi-structured citizen-science initiatives. We evaluated the resulting dataset by comparing common community diversity metrics in our work to a dataset containing all past observations from Cedar Lake posted by citizen scientists. Our study provides a baseline for documenting the impact on biodiversity derived from the physical changes to the habitat of Cedar Lake and is relevant to urban studies around the globe that aim to document biodiversity and its changes over time.

### Methods

### Study site

**Figure 1:**
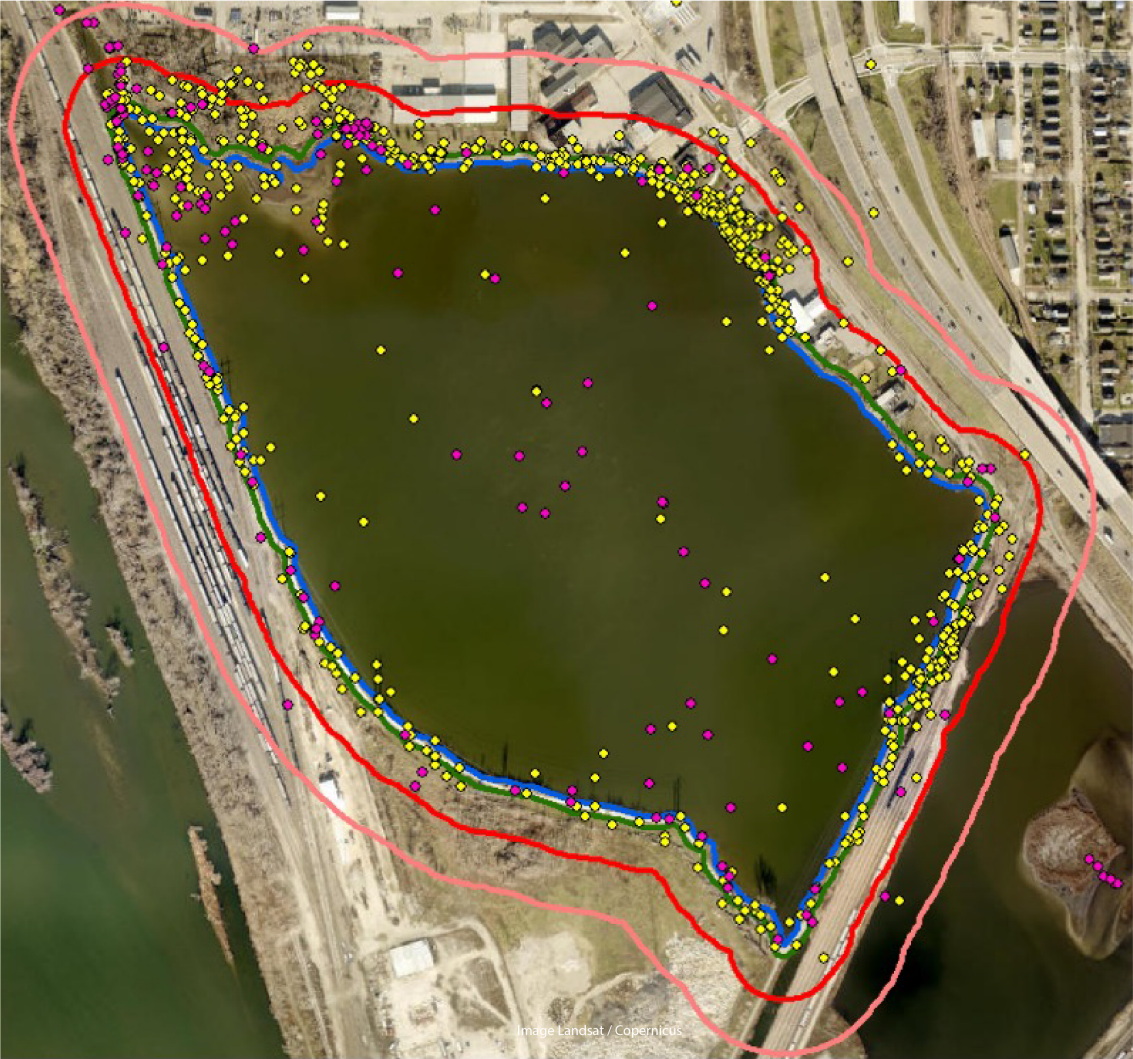
Map of study site and observations. Map of study area at Cedar Lake, Linn County, Iowa. Comparison of Research Grade observations between time periods with pink dots representing prior work (2008–2021) and yellow dots our study (2021–2022). Colored lines are lake boundaries with blue representing the lake edge (0 m), green ≤10 m from lake edge, red ≤50 m from lake edge, and pink ≤100 m from lake edge. Observations recorded beyond 100 m from the lake’s shore were excluded from analysis. Image modified from Google Earth (Image Landsat/Copernicus).

The study was conducted at Cedar Lake, Cedar Rapids, Linn County, Iowa (Fig. 1). Cedar Lake is a 0.49-km^2^ urban lake in the center of the city and currently serves as a drainage for most of the city’s waterways, a flood barrier for the nearby Cedar River, and as a recreational space for fishing, biking, and kayaking. The climate in Cedar Rapids is characterized by hot summers and cold winters, with monthly average normal temperatures ranging from −5.9 °C in January to 23.6 °C in July (National Oceanic and Atmospheric Administration, 2023). The shoreline is dominated by a mix of vegetation types and urban features, including trees, marshes, buildings, rocks, and paved walkways. From anecdotal observations, we have found that the lake supports a variety of plant species, including emergent species such as cattails and bulrushes, submergent species like pondweed and coontail, and floating species such as duckweed. To our knowledge, the biodiversity of Cedar Lake has never been formally assessed because no surveys have ever been published from there, thus available information concerning almost any aspect of Cedar Lake’s resident biota is nonexistent in the peer-reviewed literature.

### History of Cedar Lake

Cedar Lake was created in the late 1800s as a reservoir to provide drinking water for the city of Cedar Rapids. In the 1900s, the lake experienced significant environmental degradation, with pollution and sedimentation reducing water quality and harming aquatic life to the point that the health department had a yearly task of clearing dead fish from the lake. In 1909, the northern part of the lake was purchased by an electric company who boasted that they would make the lake beautiful (Gazette, 2013a). In 1912, a railroad company filled part of the lake and added a roadway with multiple rail tracks on top of it. This addition of highly used rail tracks seemed to have the effect of killing many fish, thereby creating a foul smell that citizens thought polluted the water supply. In 1939, work began on a 10-year project to clean up the lake and eliminate the foul odor, which included covering the surface with cinder and ashes, as well as treating the water with a nitrate compound. In 1979, a community committee allocated funds to renovate the lake, and three years later a power company that owned the majority of the lake, which it actively used to cool equipment, agreed to lease the lake and its shoreline to the city. Shortly after, 0.06 km^2^ of the shoreline was purchased in order to build a walking path around the lake. In 1986, the Iowa Department of Natural Resources (IDNR) issued a fish-consumption advisory because of the high number of pollutants found in tissues of fish from Cedar Lake, and the city government discouraged swimming in the lake due to this proclamation. In 1990, chromium was found to have leaked into the lake, so fishing was banned (Gazette, 2013b). In 2015, IDNR removed Cedar Lake from its Impaired Waters List, however, swimming remained discouraged. In 2019, the city of Cedar Rapids purchased the North Cell of Cedar Lake from a power company with the intention of converting the lake into recreational space (Morelli, 2019). Proposed transformations to improve the lake included mitigation of stormwater runoff *via* local drainages and dealing with the extensive silt buildup and potential toxins. These renovations began at the end 2021 and are expected to be finished by 2025 (Payne, 2021).

### Citizen science initiatives

To document the biodiversity of Cedar Lake, we used iNaturalist (http://www.inaturalist.org), which is a global citizen science initiative created by the California Academy of Sciences and the National Geographic Society, with over 89 million observations around the world as of 1 February 2022. We created a project on iNaturalist (https://www.inaturalist.org/projects/the-biodiversity-of-cedar-lake) with parameters to include observations (*i.e.,* photo-vouchered observations) of all organisms in Linn County, Iowa, by individuals who joined the project (*i.e.,* observers) during the active season of 2021. We ran the project for a full calendar year 1 April 2021 to 31 March 2022 to allow time for community identifications of our observations, at which point we downloaded our project’s data. Since we were primarily interested in biodiversity during the active season, we note that our project did not cover winter (November to March), thus this seasonal period was not included in the resulting dataset. On iNaturalist, observers upload photos of organisms and users attempt to identify the organism to the lowest possible taxonomic level. Community members of iNaturalist can confirm or deny identifications of observations, resulting in three levels of confirmation: (1) “Research Grade”, which has been confirmed by at least two different individuals; (2) “needs ID”, which includes observations not yet identified by two individuals; (3) and “casual”, which includes observations that are of low quality or lack specificity.

To engage the local community, we held 12 BioBlitz events at Cedar Lake bi-weekly from April through October of 2021 (Meeus et al., 2023). Each event lasted for four hours for a total of 48 h of semi-structured community surveys. At these events, community members were debriefed (Rokop et al., 2022) during which they were encouraged to take photos of all plants and animals (alive, dead, or animal signs), and upload these observations to our project on iNaturalist (The Biodiversity of Cedar Lake). We started each event with brief instructions on how to take biodiversity observations, how to avoid duplicates, how to upload them to iNaturalist, and how to join our project on the website. We also explicitly told individuals who attended the initial introductions that we were interested in photos of all animals and plants from anywhere near the lake, including off the main walking trail. We created a website (https://www.thebiodiversityofcedarlake.com/) that included tutorials on how to use iNaturalist, dates and times of our BioBlitz events, and links to the project page on iNaturalist. We also promoted our BioBlitz events with a Facebook page (https://www.facebook.com/TheBiodiversityofCedarLake), which we shared through emails to the entire Coe College campus (Forti, 2023). Marissa Payne from The Gazette, a local newspaper, authored an article (https://www.thegazette.com/local-government/coe-college-team-spearheads-cedar-rapids-community-based-project-to-study-cedar-lake-biodiversity/) about our project, that also helped to increase awareness in the Cedar Rapids area. We note that both authors (AA and DFH) were active participants in the data collection process at the BioBlitz events.

To understand the impact of citizen-science initiatives on the documentation of biodiversity at Cedar Lake, we used standard biodiversity metrics (see ‘Statistical analyses’) to compare our data to all previous iNaturalist observations recorded at Cedar Lake from the inception of iNaturalist in 2008 to the start of our project in 2021.

### Spatial analyses

We used ArcGIS *v.* 10.1 to map the spatial distribution of iNaturalist observations in both the prior dataset and our study. We created buffers in ArcGIS around Cedar Lake to pool samples for comparison between time periods using distances: 0 m, ≤ 10 m, ≤ 50 m, and ≤ 100 m intervals. We excluded observations from the following statistical analyses that were >100 m from the lake’s shoreline.

### Statistical analyses

We used Microsoft Excel 2016 (Redmond, WA, USA) to organize data and R (R Core Team, 2022) with the RStudio interface (PositTeam, 2022) for statistical analyses. We used only Research Grade observations recorded within a 100 m buffer from the lake’s shore to compare our study to all previous observations on iNaturalist recorded at Cedar Lake. We used a variety of commonly used species diversity metrics to compare the biological community between our dataset and prior data recorded at the site. We used the R package Codyn (Hallett et al., 2016) to calculate the community metrics Shannon Diversity Index and Simpson’s Evenness for both datasets. We used the approach developed by Hutcheson (1970) to statistically compare species diversity indices between datasets (Zar, 2009). We also used the R package Codyn to generate rank-frequency curves for each dataset to assess how the species distribution rank differed between time periods (Avolio et al., 2019). The rank frequency curves were analyzed with Codyn to compare various aspects of the curves between time periods such as differences in species richness, evenness, rank, composition, and overall curve difference. We used the R package iNEXT (Hsieh, Ma & Chao, 2016) to calculate species rarefaction curves with extrapolation using 1,000 bootstrap replicates for both datasets. We used rarefaction curves to compare the rate of increase in the number of species between the two datasets relative to the number of individuals observed (Roswell, Dushoff & Winfree, 2021). We also used the R package iNEXT to estimate species diversity using the Chao richness method that we compared the overlap between 95% confidence intervals between for each dataset (Chao & Chiu, 2016).

## Results

Our iNaturalist project (The Biodiversity of Cedar Lake) went online 1 April 2021, and the first observation was uploaded on 4 April 2021 and the last on 18 October 2021, which corresponds to the active growing season in eastern Iowa. During this period, we recorded a total of 1,345 biodiversity observations with 787 of these becoming Research Grade observations from 60 different observers by 31 March 2022 when we downloaded the data for analysis (Table 1). For these observations, 232 species were detected, 200 of which were classified as Research Grade. In the prior dataset from this site, the first observation was uploaded on 6 July 2011 and the last on 25 March 2021. During this period, a total of 257 biodiversity observations were uploaded with 168 of these becoming Research Grade observations from 22 observers. For these observations, 182 species were detected, 86 of which were classified as Research Grade. We found that most Research Grade observations (>90%) were recorded from within 100 m of the lake’s shore in both datasets (Fig. 1; Table 2). Only 41 species were shared between the two time periods, with 51 species unique to the prior dataset (mostly birds) and 159 to our work (mostly insects and plants). The top observer in the prior dataset contributed 43% (72 out of 168) of the total Research Grade observations and documented 43% of the species (37 out of 86), whereas the top observer in our work contributed 35% (272 out of 787) of the Research Grade observations and documented 63% of the species (125 out of 200).

**Table 1:** Comparison of iNaturalist datasets between two time periods at Cedar Lake, Linn County, Iowa, by number of Research Grade observations and species with the total number of observations and species in parentheses. Only Research Grade observations were used to estimate species richness, Shannon Diversity Index, Simpson’s Evenness, and number of observations by major group. Chao species richness estimate is presented with ± 1 standard error followed by 95% confidence interval in parentheses.

	Prior work (2008–2021)	Our study (2021–2022)
Observations	168 (257)	787 (1345)
Species	86 (182)	200 (232)
*Community metrics*		
Species Richness	174.4 ± 33.4 (129.2–267.0)	374.2 ± 48.2 (302.4–496.6)
Shannon Index	4.012793	4.510965
Evenness	0.304439	0.219352
*Observations by major group (% of total)*		
Vertebrate	132 (78.6%)	439 (55.7%)
Invertebrate	17 (10.1%)	253 (29.9%)
Plant	19 (11.3%)	95 (12.1%)
Other	–	18 (2.3%)

**Table 2:** Comparison of Research Grade observations between time periods within four boundaries of Cedar Lake, Linn County, Iowa. Percentage of the total Research Grade observations presented in parentheses.

	Prior work (2008–2021)	Our study (2021–2022)
Within lake bounds	57 (33.9%)	181 (22.9%)
≤ 10 m of lake edge	100 (59.5%)	465 (59.1%)
≤ 50 m of lake edge	145 (86.3%)	748 (95.0%)
≤ 100 m of lake edge	154 (91.6%)	780 (99.1%)

**Figure 2:**
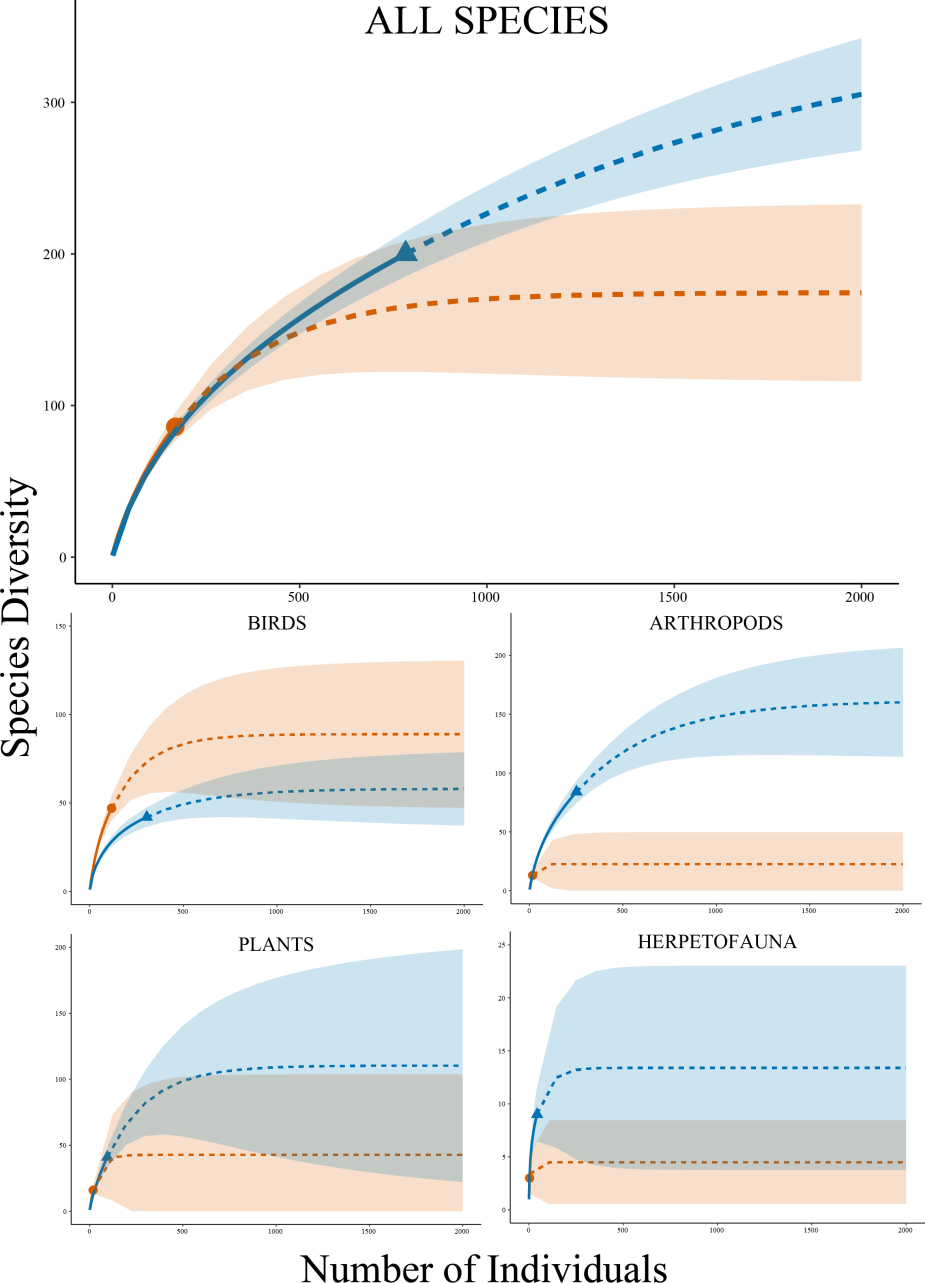
Species accumulation curves. Rarefaction species accumulation curves with extrapolation and 95% confidence intervals by group for Research Grade observations at Cedar Lake, Linn County, Iowa. Orange circle represents prior work (2008–2021) and blue triangle our study (2021–2022). Solid lines are observed and dashed lines are extrapolated values. Curves were generated with 1,000 bootstrap replicates using the R package iNEXT.

The estimated species richness using the Chao method for our study was more than double that of the prior work at the site (374 species *versus* 174 species), with an upper bound of nearly 500 species in our work compared to an upper bound of just about 275 in the prior work (Table 1). Citizens detected 200 of the 374 estimated species in our data (53.5%), compared to 86 of the 174 estimated species in the past data (49.4%). A temporal assessment where data in the prior work was restricted to the same months as our work (April to October) revealed an even wider gap between time periods for estimated species richness with the prior work down to just 131.1 ± 36.5 species (95% CI = 85.4–238.9 species). Rarefaction curves indicated that our work not only detected more species with more individuals than the prior work, but extrapolations suggested that continued efforts using our approach may result in a much higher amount of species diversity detected at the site overall (Fig. 2). The Shannon Diversity Indices for the two time periods were both >4, with our work being greater than 4.5, indicating that we detected significantly more species from a wider range of abundances than the past data (*t* = 19.57, *df* = 188, *P* < 0.001). Simpson’s Evenness values showed that the past data detected a more even community (0.3 *versus* 0.22). Our work demonstrated a much more balanced distribution of observations among major groups of biodiversity—vertebrates (56%), invertebrates (30%), and plants (12%)—whereas the prior work was less balanced because it was dominated by vertebrate observations (>75%). An assessment of college involvement where we excluded the authors’ iNaturalist contributions resulted in a species richness estimate for our data of 249.9 ± 39.5 species (95% CI = 193.7–354.6 species), which produced similar diversity indices between the datasets (*t* = 0.39, *df* = 226, *P* = 0.69).

Analyses by taxonomic groups found similar patterns to the full dataset with several important distinctions, including the fact that the observed species accumulation curves did not reach asymptotes. For arthropods (phylum Arthropoda), rarefaction curves estimated higher species diversity with non-overlapping confidence intervals in our dataset compared to the past work (Fig. 2), which was supported by a significantly higher Shannon Diversity Index in our data (3.94 *versus* 2.51) (*t* = 9.83, *df* = 17, *P* < 0.001). Higher diversity in our work was also projected by curves for plants (kingdom Plantae) with a higher diversity index (3.05 *versus* 2.73) (*t* = 2.21, *df* = 21, *P* = 0.039) and herpetofauna (classes Amphibia and non-avian Reptilia) also with a higher diversity index (1.82 *versus* 1.04) (*t* = 3.17, *df* = 4, *P* = 0.034), but both had overlapping confidence intervals for extrapolated data. Birds (class Aves) were the sole exception with slightly higher richness estimates in the past data and a higher diversity index (3.34 *versus* 2.77) (*t* = 18.24, *df* = 145, *P* < 0.001), but confidence intervals also overlapped in this analysis. Estimated species richness using the Chao method by these four major taxonomic groups showed similar results to the rarefaction curves and community comparisons (Table 3).

**Figure 3:**
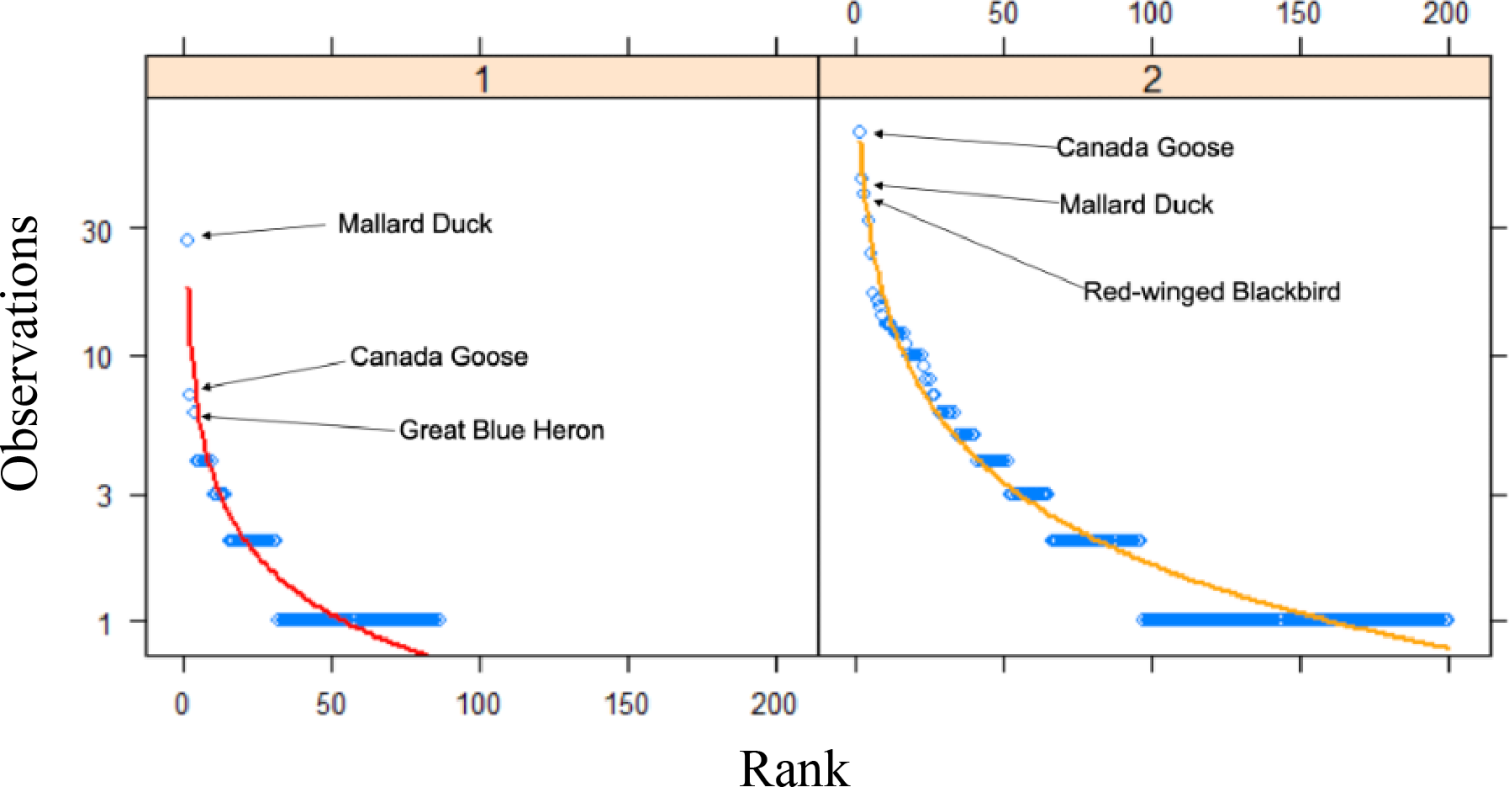
Rank curves for species between time periods. Rank frequency of observation curves for Research Grade observations at Cedar Lake, Linn County, Iowa. Red line (Zipf) represents prior work (2008–2021) and orange line (Mandelbrot) our study (2021–2022) with the top three species indicated for both.

An assessment of community composition metrics, as determined by the number of Research Grade observations, between time periods revealed that only the top two species were present in both datasets out of the top 10 most frequently observed species (Table 4). Among the top 10 most frequently observed species in the past time period, all were vertebrates, and included nine species of bird and one mammal. In comparison, the top 10 most frequently observed species in our study included four bird species, three invertebrates, two plants, and one fish. The rank frequency of observations curves between the time periods revealed that many more species in our data were detected more frequently (>10 observations) compared to the past data, which only had a single species detected >10 times (Fig. 3). For example, the top two species in the past data represented 20.2% of all observations in that dataset, whereas the same top two species made up 14.6% of all observations in our data. Quantitative differences between the rank frequency curves for the two time periods detected increases in our data relative to the past data for species richness (0.465), species rank (0.279), species composition (0.367), and overall curve difference (508.6), with only a single decrease which was detected for evenness (−0.245).

## Discussion

Datasets built by citizen scientists will be important to understanding how global environmental changes, such as urbanization, will impact biodiversity worldwide. At a single site, we compared opportunistic citizen-science observations posted on iNaturalist (2008–2021) to semi-structured observations (*i.e.,* BioBlitz events) posted over the course of a single active season (April–October 2021), equating to 48 h of directed surveys. In comparison to historical observations at Cedar Lake, our baseline data provided a clear improvement to our understanding of the biodiversity in and around the site. Below we discuss potential biases impacting citizen-science studies with illustrative examples from our own efforts. Ultimately, we emphasize the need for ongoing monitoring that uses systematic data collection for citizen science coupled with structured surveys and complementary census techniques to track future changes in biodiversity at this urban lake.

**Table 3:** Comparison of Research Grade observations for four major taxonomic groups between two time periods at Cedar Lake, Linn County, Iowa. Chao species richness estimate is presented with ± 1 standard error followed by 95% confidence interval in parentheses.

	Prior work (2008–2021)	Our study (2021–2022)
Birds		
Observations	117	305
Observed Species	47	42
Species Richness	88.9 ± 23.1 (62.3–161.8)	58.0 ± 11.0 (46.7–96.2)
Arthropods		
Observations	17	252
Observed Species	13	84
Species Richness	22.5 ± 8.5 (15.1–55.8)	161.6 ± 32.8 (119.0–255.7)
Plants		
Observations	19	95
Observed Species	16	41
Species Richness	42.7 ± 21.9 (22.5–125.4)	110.4 ± 39.2 (65.7–235.5)
Herpetofauna		
Observations	4	44
Observed Species	3	9
Species Richness	4.5 ± 2.9 (3.1–20.1)	13.4 ± 7.0 (9.5–49.0)

**Table 4:** Ten most frequently observed species represented in both datasets from Cedar Lake, Linn County, Iowa, presented with the number of Research Grade observations per species. Species in bold are shared between top 10 lists.

	Prior work (2008–2021)	Our study (2021–2022)
**1**	**Mallard (** ***Anas platyrhynchos*** **) = 27**	**Canada Goose (** ***Branta canadensis*** **) = 69**
**2**	**Canada Goose (** ***Branta canadensis*** **) = 7**	**Mallard (** ***Anas platyrhynchos*** **) = 46**
3	Great Blue Heron (*Ardea herodias*) = 6	Red-winged Blackbird (*Agelaius phoeniceus*) = 40
4	Ruddy Duck (*Oxyura jamaicensis*) = 4	American Robin (*Turdus migratorius*) = 32
5	White-tailed Deer (*Odocoileus virginianus*) = 4	Great Mullein (*Verbascum thapsus*) = 24
6	Lesser Scaup (*Aythya affinis*) = 4	Common Eastern Bumblebee (*Bombus impatiens*) = 17
7	Green-Winged Teal (*Anas carolinensis*) = 4	Differential Grasshopper (*Melanoplus differentialis*) = 16
8	Ring-billed Gull (*Larus delawarensis*) = 4	Bluegill (*Lepomis macrochirus*) = 15
9	American White Pelican (*Pelecanus erythrorhynchos*) = 4	Cabbage White (*Pieris rapae*) = 14
10	American Kestrel (*Falco sparverius*) = 3	Purple Crownvetch (*Securigera varia*) = 13

Our approach engaged more community members and produced higher biodiversity metrics than was found throughout several years of opportunistic observations recorded at Cedar Lake. Our findings showed that adding structure to citizen-science activities, coupled with digital biodiversity tools and college-student involvement, can generate more data than passive approaches, even over short timescales (Kelling et al., 2019). Several diversity metrics in our dataset were higher than in the prior work, indicating that significantly more biodiversity is present in the area than could have been realized with the past data alone. Our efforts, however, are still likely a vast underestimation of the true species diversity of Cedar Lake as nearly all observed species accumulation curves failed to reach an asymptote and citizen scientists observed just slightly more than half of the estimated species richness. Consequently, additional sampling that incorporates multiple techniques (Christie et al., 2019) in addition to spatially and temporally structured surveys (Kamp et al., 2016) is needed to produce a more complete biodiversity estimate of the site. Nonetheless, including semi-structured initiatives in citizen-science studies led by a local college appear to generate biodiversity assessments that are closer to an accurate representation of the community at a site over passive, opportunistic datasets (Gigliotti, Franzem & Ferguson, 2023). Because both datasets were not collected systematically, however, it remains unclear if at least some of the differences could be attributed to annual variation in species occupancy, variation in time frames, number of observers, or even preferences of observers. For example, some locations around the lake were apparently easier for observers to document biodiversity from, such as along the main trail (Fig. 1), which could be remedied in future studies with additional training at the outset of surveys. Lastly, we acknowledge that the contribution from the local college—where both authors were employed—cannot be overlooked as citizen involvement in isolation would not have produced the same diversity metrics, thus higher education can be an important catalyst in societal efforts to track local species.

The past opportunistic data from Cedar Lake on iNaturalist did exhibit a more even community than our dataset, however, all other diversity metrics were greater in our work. The greater evenness was because more species were observed only once in the past data (64% were singletons) compared to our work (52% were singletons). Regardless, several aspects of the community composition captured in the past data suggested that opportunistic approaches to biodiversity monitoring with citizen scientists are plagued with biases, often associated with a lack of formal ecological training, that may render such datasets difficult to use (Courter et al., 2013; Kamp et al., 2016; Callaghan et al., 2020). For example, nearly 70% of the Research Grade observations and 55% of the species in the past dataset were of birds, whereas birds represented about 49% of the observations and just 21% of the species in our data. Furthermore, analyses indicated that bird diversity was higher in the past data compared to our work, which is likely due to missing migratory or otherwise uncommon species. For example, the past data included 14 more duck species (family Anatidae) than our data (17 *versus* three species), most of which were winter migrants whose temporal occupancy of the lake would not have overlapped with our sampling period. Birds are often the most conspicuous vertebrates in many habitats and these results suggest that unstructured citizen-science observations tend to be biased towards such taxonomic groups (Horns, Adler & Şekercioğlu, 2018; Callaghan et al., 2021). Less conspicuous vertebrate groups, such as herpetofauna, tend to receive much less attention unless surveyors are explicitly targeting these taxa (Troudet et al., 2017), an observation that is borne out in our data. Less than 2.5% of observations in the past data were of reptiles and amphibians, which included just three species, whereas these groups represented more than 5% of the observations in our data and included nine species. Despite the popularity of fishing at Cedar Lake, only a single observation of one fish species was recorded in the past data compared to 57 observations of 14 species in our data. One of the largest increases in biodiversity representation for animals derived from our semi-structured citizen-science activities were found among the invertebrates, especially mollusks and arthropods. For example, not a single mollusk observation was recorded in the past data, compared to 10 in our work (albeit of a single species). Similarly, among the arthropods, class Insecta was just 10% of the past observations compared to this group representing nearly 30% of our observations. Plants were similarly represented in terms of proportion of overall observations in both datasets. Nevertheless, only 16 species of plants were detected in the past data compared to 41 in our data. Overall, directed activities incorporated into citizen-science surveys appear to facilitate improved biodiversity data compared to passive observations as they may help reduce some of the biases inherent to citizen science (Stevenson, Merrill & Burn, 2021), especially related to the tendency of individuals to overemphasize observations of conspicuous taxonomic groups (Mair & Ruete, 2016; Troudet et al., 2017).

**Figure 4:**
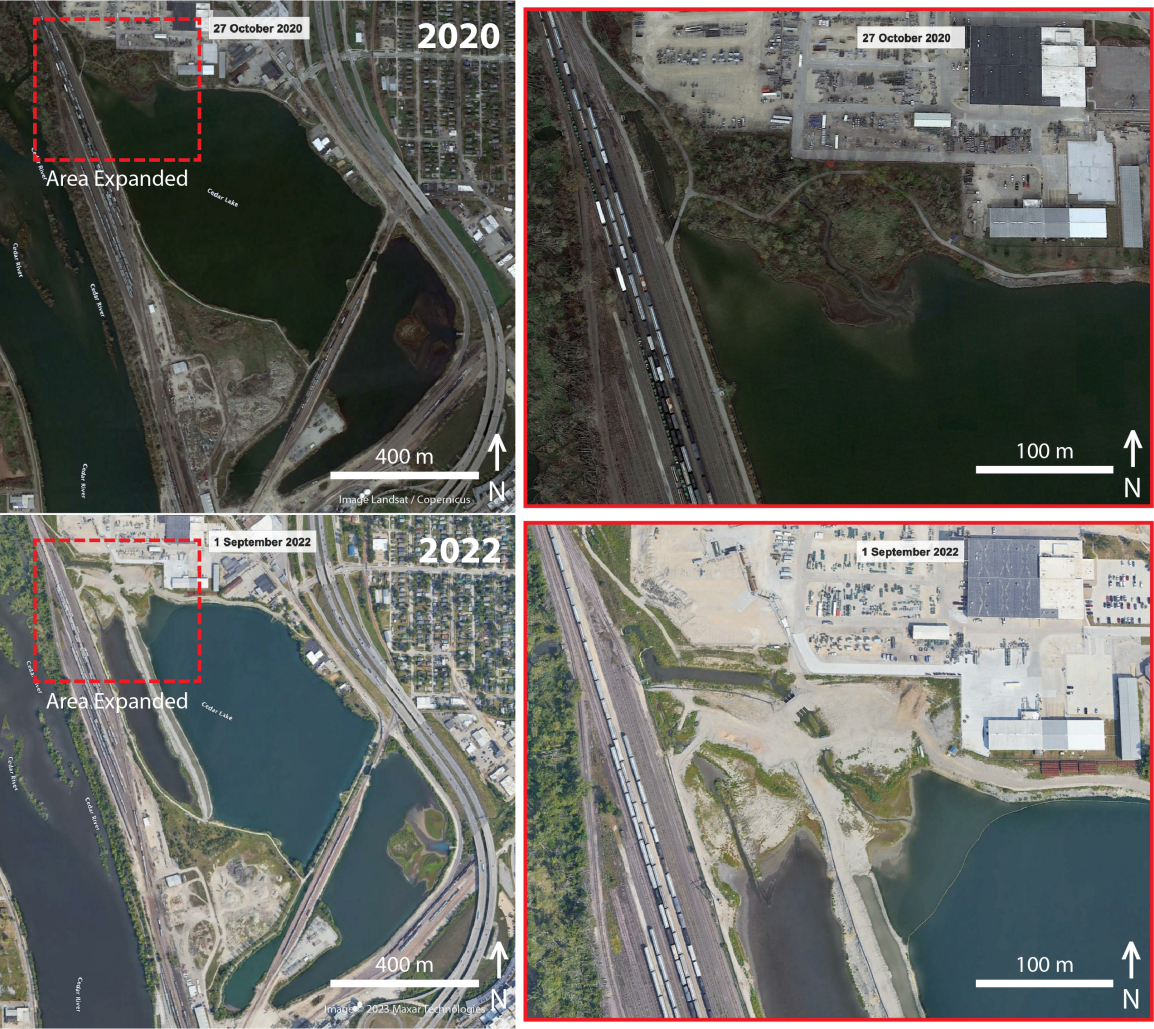
Before and after construction from satellite imagery. Overhead satellite images of the study site at Cedar Lake, Linn County, Iowa, taken before (2020) and after (2022) major structural changes began in an effort to renovate the site for flood prevention and improvement of recreational services. Our surveys took place in 2021 before habitat alterations were underway, thus serving as a critical baseline for biodiversity at this site. Images modified from Google Earth: Top (Image Landsat/Copernicus); Bottom (Image©2023 Maxar Technologies).

In 2019, the city of Cedar Rapids and ConnectCR articulated master plans (https://connectcr.org/cedar-lake-master-plan) to convert Cedar Lake from an industrial cooling site into a recreational hub through improvements to address water quality, including mitigating stormwater runoff, addressing sediment buildup, installing flood control features, and adding new trails and bridges (Morelli, 2019). Other suggested improvements for Cedar Lake included accessible boat launches, fishing piers, an obstacle course, enhanced fishing amenities, a boardwalk over the lake, and an enhanced wetland area (White, 2021). Plans to transform the lake were carried out under provisions to assess the quality of the water with respect to studying the lake floor sediment for toxins (Morelli, 2023) and redirecting flow regimes (Iowa Department of Natural Resources, 2022). Moreover, an environmental assessment of Cedar Lake included in the Cedar Rapids flood risk management project concluded a finding of “no significant impact”, with most biodiversity concerns focused on four endangered species that may have occurred in the region and the productivity of altered wetlands for aquatic species (US Army Corps of Engineers, 2019). The groundbreaking ceremony for this renovation occurred 7 October 2021, around when our project was completed. Using the time tool on Google Earth, we were able to visualize clear physical changes to habitats contiguous with the lake in satellite imagery nearly one year after the groundbreaking ceremony (1 September 2022) (Fig. 4). In particular, construction activities in the northwest corner of Cedar Lake resulted in the removal all woody vegetation in the area, the production of a 2,200 ft levee, and a complete alteration the lake’s outflow through McLoud Run, a creek that serves as drainage for the lake (Fig. 4, inset). These satellite images were captured to show that changes to the lake began after our project, and they can be used by future researchers to document spatial variation in biodiversity changes in relation to such habitat changes. It remains unknown what long-term biodiversity impacts these significant structural changes will have, but such urban land-use changes generally decrease non-avian vertebrate diversity while increasing plant diversity from the importation of non-native species (McKinney, 2008). For birds and arthropods, urban modifications often reduce richness and diversity, but increase abundance due to the dominance of a few synanthropic species (McKinney, 2008; Faeth, Bang & Saari, 2011).

At the moment, we do not know the resilience of the local species we documented, nor how they will respond to changes such as a new hydrological regime, and we know very little about their ability to migrate to other areas or even recolonize the lake while its habitats regenerate. However, we can leverage the baseline data herein, combined with the historical data, to predict what changes may occur to the biodiversity of Cedar Lake and then conduct follow-up studies at different time intervals post construction to test whether survey data support or reject such predictions. We note that management plans to modify natural or even semi-natural areas could be improved by conducting more rigorous preliminary surveys before construction to establish the baseline data needed to monitor changes over time and to determine potential impacts on resident biodiversity (Underwood, Francis & Gerber, 2011; Rojas et al., 2022), many of the consequences of such changes can be analyzed in the future. Ultimately, without reliable baseline data collected before major disturbances, it could never be possible to understand such impacts on biodiversity, let alone test predictions about what could happen in the future.

## Supplemental Information

10.7717/peerj.17209/supp-1 Supplemental Information 1 R script for biodiversity analyses Data sets made from the raw iNatualist output for both time periods can be analysed with this code to replicate the analyses in the study.

10.7717/peerj.17209/supp-2 Supplemental Information 2 Raw data for both time periods Includes only Research Grade observations from within 100 m of the lake’s shore

## Additional Information and Declarations

### Data Availability

The following information was supplied regarding data availability: The R Script for analyzing community metrics and raw data from iNaturalist for both time periods are available in the Supplemental File.

## References

[ref-1] Avolio ML, Carroll IT, Collins SL, Houseman GR, Hallett LM, Isbell F, Koerner SE, Komatsu KJ, Smith MD, Wilcox KR (2019). A comprehensive approach to analyzing community dynamics using rank abundance curves. Ecosphere.

[ref-2] Bobrowiec PED, Tavares VDC (2017). Establishing baseline biodiversity data prior to hydroelectric dam construction to monitoring impacts to bats in the Brazilian Amazon. PLOS ONE.

[ref-3] Butchart SH, Walpole M, Collen B, Van Strien A, Scharlemann JP, Almond RE, Baillie JE, Bomhard B, Brown C, Bruno J, Carpenter KE (2010). Global biodiversity: indicators of recent declines. Science.

[ref-4] Callaghan CT, Ozeroff I, Hitchcock C, Chandler M (2020). Capitalizing on opportunistic citizen science data to monitor urban biodiversity: a multi-taxa framework. Biological Conservation.

[ref-5] Callaghan CT, Poore AG, Hofmann M, Roberts CJ, Pereira HM (2021). Large-bodied birds are over-represented in unstructured citizen science data. Scientific Reports.

[ref-6] Chandler M, See L, Copas K, Bonde AM, López BC, Danielsen F, Legind JK, Masinde S, Miller-Rushing AJ, Newman G, Rosemartin A (2017). Contribution of citizen science towards international biodiversity monitoring. Biological Conservation.

[ref-7] Chao A, Chiu CH (2016). Species richness: estimation and comparison. Wiley StatsRef: Statistics Reference Online.

[ref-8] Chapman MG (2003). Paucity of mobile species on constructed seawalls: effects of urbanization on biodiversity. Marine Ecology Progress Series.

[ref-9] Christie AP, Amano T, Martin PA, Shackelford GE, Simmons BI, Sutherland WJ (2019). Simple study designs in ecology produce inaccurate estimates of biodiversity responses. Journal of Applied Ecology.

[ref-10] Costello MJ, Wieczorek J (2014). Best practice for biodiversity data management and publication. Biological Conservation.

[ref-11] Courter JR, Johnson RJ, Stuyck CM, Lang BA, Kaiser EW (2013). Weekend bias in Citizen Science data reporting: implications for phenology studies. International Journal of Biometeorology.

[ref-12] Daniels T (2001). Smart growth: a new American approach to regional planning. Planning Practice and Research.

[ref-13] Ewing R, Kostyack J, Chen D, Stein B, Ernst M (2005). Endangered by Sprawl. How runaway development threatens America’s Wildlife.

[ref-14] Faeth SH, Bang C, Saari S (2011). Urban biodiversity: patterns and mechanisms. Annals of the New York Academy of Sciences.

[ref-15] Feest A (2006). Establishing baseline indices for the quality of the biodiversity of restored habitats using a standardized sampling process. Restoration Ecology.

[ref-16] Forti LR (2023). Students as citizen scientists: project-based learning through the iNaturalist platform could provide useful biodiversity data. Biodiversity.

[ref-17] Gazette CR (2013a). ‘The Slough’, aka Cedar Lake. https://www.thegazette.com/history/the-slough-aka-cedar-lake.

[ref-18] Gazette CR (2013b). Cedar Lake: industry vs. recreation. https://www.thegazette.com/history/cedar-lake-industry-vs-recreation/.

[ref-19] Gigliotti FN, Franzem TP, Ferguson PF (2023). Rapid, recurring, structured survey versus bioblitz for generating biodiversity data and analysis with a multispecies abundance model. Conservation Biology.

[ref-20] Gullison RE, Hardner J, Anstee S, Meyer M (2015). Good Practices for the Collection of Biodiversity Baseline Data. Prepared for the Multilateral Financing Institutions Biodiversity Working Group & Cross-Sector Biodiversity Initiative.

[ref-21] Hallett LM, Jones SK, MacDonald AAM, Jones MB, Flynn DF, Ripplinger J, Slaughter P, Gries C, Collins SL (2016). Codyn: an r package of community dynamics metrics. Methods in Ecology and Evolution.

[ref-22] Hanford JK, Webb CE, Hochuli DF (2020). Management of urban wetlands for conservation can reduce aquatic biodiversity and increase mosquito risk. Journal of Applied Ecology.

[ref-23] Hassall C (2014). The ecology and biodiversity of urban ponds. Wiley Interdisciplinary Reviews: Water.

[ref-24] Hill MJ, Biggs J, Thornhill I, Briers RA, Gledhill DG, White JC, Wood PJ, Hassall C (2017). Urban ponds as an aquatic biodiversity resource in modified landscapes. Global Change Biology.

[ref-25] Horns JJ, Adler FR, Şekercioğlu ÇH (2018). Using opportunistic citizen science data to estimate avian population trends. Biological Conservation.

[ref-26] Hsieh TC, Ma KH, Chao A (2016). iNEXT: an R package for rarefaction and extrapolation of species diversity (Hill numbers). Methods in Ecology and Evolution.

[ref-27] Hutcheson K (1970). A test for comparing diversities based on Shannon formula. Journal of Theoretical Biology.

[ref-28] Iowa Department of Natural Resources (IDNR) (2022). Cedar Lake dewatering rational. National Pollutant Discharge Elimination System. https://programs.iowadnr.gov/wwpie/Admin/PermitDetails?permitID=8323.

[ref-29] Kamp J, Oppel S, Heldbjerg H, Nyegaard T, Donald PF (2016). Unstructured citizen science data fail to detect long-term population declines of common birds in Denmark. Diversity and Distributions.

[ref-30] Kelling S, Johnston A, Bonn A, Fink D, Ruiz-Gutierrez V, Bonney R, Fernandez M, Hochachka WM, Julliard R, Kraemer R, Guralnick R (2019). Using semistructured surveys to improve citizen science data for monitoring biodiversity. BioScience.

[ref-31] Kirchhoff C, Callaghan CT, Keith DA, Indiarto D, Taseski G, Ooi MK, Le Breton TD, Mesaglio T, Kingsford RT, Cornwell WK (2021). Rapidly mapping fire effects on biodiversity at a large-scale using citizen science. Science of the Total Environment.

[ref-32] Magurran AE, Baillie SR, Buckland ST, Dick JM, Elston DA, Scott EM, Smith RI, Somerfield PJ, Watt AD (2010). Long-term datasets in biodiversity research and monitoring: assessing change in ecological communities through time. Trends in Ecology & Evolution.

[ref-33] Mair L, Ruete A (2016). Explaining spatial variation in the recording effort of citizen science data across multiple taxa. PLOS ONE.

[ref-34] McKinney ML (2008). Effects of urbanization on species richness: a review of plants and animals. Urban Ecosystems.

[ref-35] Meeus S, Silva-Rocha I, Adriaens T, Brown PM, Chartosia N, Claramunt-López B, Martinou AF, Pocock MJ, Preda C, Roy HE, Tricarico E (2023). More than a bit of fun: the multiple outcomes of a bioblitz. BioScience.

[ref-36] Mihoub JB, Henle K, Titeux N, Brotons L, Brummitt NA, Schmeller DS (2017). Setting temporal baselines for biodiversity: the limits of available monitoring data for capturing the full impact of anthropogenic pressures. Scientific Reports.

[ref-37] Morelli B (2019). Cedar Rapids wants to buy Cedar Lake for $1. The Cedar Rapids Gazette. https://www.thegazette.com/news/cedar-rapids-wants-to-buy-cedar-lake-for-1/.

[ref-38] Morelli B (2023). Environmental testing of Cedar Lake a go. The Cedar Rapids Gazette. https://www.thegazette.com/government-politics/environmental-testing-of-cedar-lake-a-go/.

[ref-39] National Oceanic and Atmospheric Administration (NOAA) (2023). Monthly climate normals (1991–2020)—Cedar Rapids (1) Iowa. https://www.weather.gov/dvn/Climate_Normals.

[ref-40] Payne M (2021). Citizen-led dream to revitalize Cedar Lake coming to life as $20M Connect CR. The Cedar Rapids Gazette. https://www.thegazette.com/local-government/citizen-led-dream-to-revitalize-cedar-lake-coming-to-life-as-20m-connect-cr/.

[ref-41] Piano E, Souffreau C, Merckx T, Baardsen LF, Backeljau T, Bonte D, Brans KI, Cours M, Dahirel M, Debortoli N, Decaestecker E (2020). Urbanization drives cross-taxon declines in abundance and diversity at multiple spatial scales. Global Change Biology.

[ref-42] Pimm SL, Jenkins CN, Abell R, Brooks TM, Gittleman JL, Joppa LN, Raven PH, Roberts CM, Sexton JO (2014). The biodiversity of species and their rates of extinction, distribution, and protection. Science.

[ref-43] Posit Team (2022). http://www.rstudio.com/.

[ref-44] Qiu L, Lindberg S, Nielsen AB (2013). Is biodiversity attractive?—On-site perception of recreational and biodiversity values in urban green space. Landscape and Urban Planning.

[ref-45] R Core Team (2022). https://www.R-project.org/.

[ref-46] Rojas C, Sepúlveda E, Jorquera F, Munizaga J, Pino J (2022). Accessibility disturbances to the biodiversity of urban wetlands due to built environment. City and Environment Interactions.

[ref-47] Rokop M, Srikanth R, Albert M, Radonic C, Vincent R, Stevenson R (2022). Looking more carefully: a successful bioblitz orientation activity at an urban public university. Citizen Science: Theory and Practice.

[ref-48] Roswell M, Dushoff J, Winfree R (2021). A conceptual guide to measuring species diversity. Oikos.

[ref-49] Sala OE, Stuart Chapin FIII, Armesto JJ, Berlow E, Bloomfield J, Dirzo R, Huber-Sanwald E, Huenneke LF, Jackson RB, Kinzig A, Leemans R (2000). Global biodiversity scenarios for the year 2100. Science.

[ref-50] Savard JPL, Clergeau P, Mennechez G (2000). Biodiversity concepts and urban ecosystems. Landscape and Urban Planning.

[ref-51] Stevenson R, Merrill C, Burn P (2021). Useful biodiversity data were obtained by novice observers using iNaturalist during college orientation retreats. Citizen Science: Theory and Practice.

[ref-52] Theobald EJ, Ettinger AK, Burgess HK, De Bey LB, Schmidt NR, Froehlich HE, Wagner C, HilleRisLambers J, Tewksbury J, Harsch MA, Parrish JK (2015). Global change and local solutions: tapping the unrealized potential of citizen science for biodiversity research. Biological Conservation.

[ref-53] Troudet J, Grandcolas P, Blin A, Vignes-Lebbe R, Legendre F (2017). Taxonomic bias in biodiversity data and societal preferences. Scientific Reports.

[ref-54] Tweddle JC, Robinson LD, Pocock MJO, Roy HE (2012). Guide to citizen science: developing, implementing and evaluating citizen science to study biodiversity and the environment in the UK.

[ref-55] Underwood JG, Francis J, Gerber LR (2011). Incorporating biodiversity conservation and recreational wildlife values into smart growth land use planning. Landscape and Urban Planning.

[ref-56] U.S. Army Corps of Engineers (2019). Environmental assessment. Cedar Rapids flood risk management project Cedar Rapids, Iowa. Rock Island District, Rock Island. https://www.mvr.usace.army.mil/Missions/Flood-Risk-Management/Cedar-Rapids/.

[ref-57] White M (2021). Cedar Rapids breaks ground to revitalize Cedar Lake and connect it to Newbo and Czech Village. KWWL. https://www.kwwl.com/news/cedar-rapids/cedar-rapids-breaks-ground-to-revitalize-cedar-lake-and-connect-it-to-newbo-and-czech/article_25c42bba-27cf-11ec-85a6-bbaab74e9770.html.

[ref-58] Zar JH (2009). Biostatistical analysis.

